# An Analysis of Priorities in Developing Virtual Reality Programs for Core Nursing Skills: Cross-sectional Descriptive Study Using the Borich Needs Assessment Model and Locus for Focus Model

**DOI:** 10.2196/38988

**Published:** 2022-11-24

**Authors:** EunYoung Jeong, JunSeo Lim

**Affiliations:** 1 Department of Nursing Wonkwang University Jeonbuk Republic of Korea; 2 Research Institute of Nursing Science College of Nursing Seoul National University Seoul Republic of Korea

**Keywords:** core nursing skills, virtual reality, Borich priority formula, Locus for Focus Model, need assessment, nursing education

## Abstract

**Background:**

There are limitations to conducting face-to-face classes following the recent COVID-19 pandemic. Web-based education is no longer a temporary form of teaching and learning during unusual events, such as pandemics, but has proven to be necessary to uphold in parallel with offline education in the future. Therefore, it is necessary to scientifically organize the priorities of a learner needs analysis by systematically and rationally investigating and analyzing the needs of learners for the development of virtual reality (VR) programs for core nursing skills (CNS).

**Objective:**

This study aimed to identify the priorities of learners’ needs for the development of VR programs for CNS using the Locus for Focus Model and Borich need assessment model.

**Methods:**

The participants included nursing students in South Korea who were in their second year or higher and had taken courses in fundamental nursing or CNS-related classes. The survey took place from May 20 to June 25, 2021. A total of 337 completed questionnaires were collected. Of these, 222 were used to conduct the final analysis. The self-report questionnaire consisted of 3 parts: perception of VR programs, demand for developing VR programs, and general characteristics. The general characteristics of the participants were analyzed using descriptive statistics. To determine the priority of the demand for developing VR programs for CNS, the Locus for Focus Model and the Borich priority formula were used.

**Results:**

In all, 7 skills were identified as being of the top priority for development, including intramuscular injection, intradermal injection, tube feeding, enema, postoperative care, supplying oxygen via nasal cannula, and endotracheal suction.

**Conclusions:**

The analysis showed that nursing students generally needed and prioritized the development of VR programs for the nursing skills involving invasive procedures. The results of this study are intended to help in various practical education classes using VR programs in nursing departments, which are currently facing difficulties in teaching CNS on the web owing to COVID-19.

## Introduction

Nursing education is aimed at ensuring the provision of high-quality nursing services by identifying and responding appropriately to various needs of nursing recipients through theoretical and practical education. Regarding practical training, the Korean Accreditation Board of Nursing Education (KABONE) selected 20 basic and frequently implemented nursing skills in nursing practice as core nursing skills (CNS). CNS are the essential nursing skills that nurses must possess. Moreover, it is recommended to instill these skills in nurses by training them using the theoretical background related and in addition to the skills [1].

Traditional core nursing practice training in nursing schools is conducted in a way that enables students to directly practice the relevant skills face to face in the practice room using models and others after receiving theoretical and demonstrative education from an educator, such as a professor or practical instructor, who is proficient in technical skills. However, there have been limitations to conducting face-to-face classes following the COVID-19 pandemic. Consequently, web-based classes using webcams have been activated. Web-based education, which is currently being implemented at many universities, is no longer a temporary form of teaching and learning during unusual events, such as pandemics, but has been proven to be necessary to uphold in parallel with offline education in the future [2]. Unlike web-based theoretical education, web-based education on CNS is limited in that students learn nursing skills and improve their actual clinical performance by watching videos and learning about the skills. Therefore, there is a pressing need to develop high-level educational tools that can be provided to students learning on the web.

To overcome the limitations of core nursing practice training conducted through web-based education, a system similar to the actual clinical field in virtual reality (VR) could be built. VR provides a more optimized and immersive learning experience as well as a self-directed and practical learner-centered learning environment for individual learners, allowing them to learn without temporal and spatial limitations in a virtual environment [3]. However, neither educators nor learners are accustomed to using cutting-edge VR technology and equipment; therefore, it is not very common in education. Developers are also not actively introducing or investing in new technologies owing to the economic burden of purchasing expensive development equipment, rapid technological development, and uncertainty in commercial feasibility [4].

In health care and medical fields, it was difficult to find studies on 3D VR–related educational content before 2005. However, active research has been conducted since 2006, with 10 studies published in 2008 and 2010. A total of sixty-two 3D VR–related studies were published in the health care and medical field from 1990 to 2013 [5], of which studies on health care and medical education accounted for the biggest proportion with 34 (55%) studies; 23 (68%) of the 34 studies were conducted on nursing-related 3D VR. 3D VR educational content used in nursing education is based on themes, such as disaster-related scenarios, postpartum bleeding simulation, and nutrition, rather than CNS, as well as focusing on the scenario-oriented simulation education field to manage “situations” that may occur in the nursing field [5]. A systematic literature review including studies on nursing education using 3D VR targeting published up to November 2018 reported that there were no studies conducted in South Korea [6]. Moreover, 4 (57%) studies, more than half of the 7 studies, were conducted in the United States. Only 2 (29%) studies used 3D VR educational content while offering induction catheterization technology education. The remaining 5 (71%) were analyzed as 3D VR simulation education using scenarios [6].

The development of educational programs begins with conducting a learning needs analysis (LNA) of learners who are end users. Diagnosing and analyzing the exact needs of learners determines participation in adult education and is an essential procedure for verifying the need for education beforehand [7]. An LNA also provides educators with the information they need to develop, plan, and implement educational strategies and achieve the educational goals of their institution [8]. Therefore, it is necessary to scientifically organize the priorities of an LNA by systematically and rationally investigating and analyzing the needs of learners for the development of VR programs for CNS. An LNA is aimed at investigating the difference between “what should be” and “what is,” analyzing the priority according to the difference, determining the priority, and finding an optimal solution [9]. Therefore, this study aimed to identify the top priority in the development of VR programs for CNS using LNA prioritization methods—such as the 2-tailed *t* test, Borich priority formula, and the Locus for Focus Model (LFM)—together and to suggest implications for the development of VR programs for CNS, hence improving educational outcomes through systematic prioritization.

## Methods

### Study Aim and Design

This cross-sectional descriptive study aimed to identify development priorities by analyzing the development needs for CNS-related VR programs for nursing students.

### Participants

The participants of this study included nursing students in South Korea who were in their second year or higher and had taken courses in fundamental nursing or CNS. The survey took place from May 20 to June 25, 2021. A total of 337 copies of the relevant questionnaire were collected. Of these, 222 (66%) copies were used to conduct the final analysis; participants who did not have experience of a VR program and those who gave blank answers were excluded.

### Instrument

#### Questionnaire Development

The self-report questionnaire comprised items on the perception of VR programs, VR program development requirements, and the general characteristics of the participants. The author first composed a draft questionnaire based on the literature review and further formed an expert group based on the criteria of Lynn [10] to verify content validity. The expert group comprised 5 professors from the nursing department who either lectured on CNS, such as fundamental nursing, or had experience in VR-related research. The expert group independently evaluated the content validity index (CVI) of the preliminary items. The content validity coefficients of each item (Item-CVI [I-CVI]) were measured using a 4-point scale; the responses ranged from 1 (“not necessary at all”) to 4 (“very necessary”). Regarding the expert opinions, items with an I-CVI of 0.80 or higher were selected, whereas those with an I-CVI less than 0.80 were removed from the questionnaire. Based on the expert opinions that some questions needed to be further subdivided, the questionnaire was modified to comprise 59 items. The draft questionnaire was then used in a preliminary survey targeting 10 nursing students. Based on their feedback, some phrases were modified in the final questionnaire.

#### Perception of VR Programs

The questionnaire on the perception of VR programs comprised 10 items, including items inquiring how much the individuals knew about the concept of a VR program, the necessity of introducing a VR program in the nursing department, and the need for developing VR programs. The answers ranged from 1 point (“strongly disagree”) to 5 points (“strongly agree”) and were scored on a 5-point Likert scale. In this study, Cronbach α was .78.

#### Needs Assessment for Developing VR Programs

In this research, the “needs” means the discrepancy between participants’ current status and future importance required to develop the VR program for CNS. Performance competence level (PCL), reflecting the current importance level, and required competence level (RCL), reflecting the priority for developing VR programs, were surveyed for 20 CNS suggested by the KABONE. Answers were given on a 5-point Likert scale, ranging from 1 (“strongly disagree”) to 5 (“strongly agree”). In this study, Cronbach α was .82.

#### General Characteristics

The questionnaire on the general characteristics of the participants comprised 9 items, including the total credits for the CNS, age, sex, etc.

### Data Analysis

The collected data were coded and analyzed using SPSS statistics software (version 25.0; IBM Corp). The general characteristics of the participants were analyzed using descriptive statistics, such as frequencies and percentages, as well as means and SDs. To determine the priority of the demand for developing VR programs for CNS, the analysis method of Cho [9] was applied as follows.

First, the significance of the average difference between the PCL and RCL was analyzed using a 2-tailed *t* test.

Second, Borich priority was calculated to determine the priority of the development of VR programs of CNS [11]. The Borich priority formula is as follows:




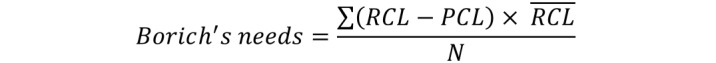




RCL is each individual’s current importance level of CNS score, PCL is each individual’s importance level for developing VR programs of CNS score, 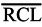 is the average of the required level, and N is the total number of cases.

**Figure 1:**
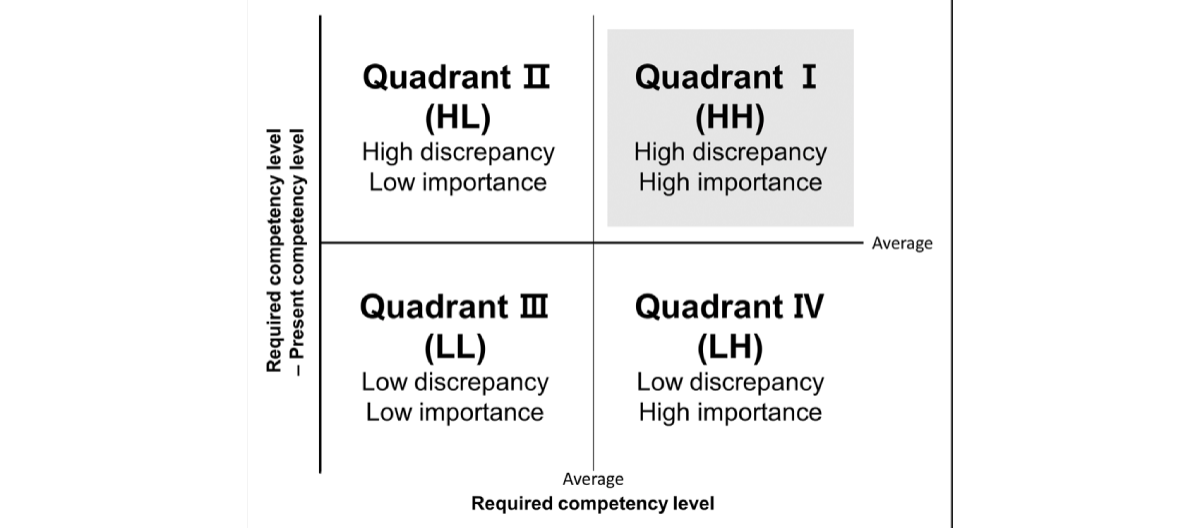
The Locus for Focus Model.

Third, priorities were visualized using the LFM. The items belonging to the first quadrant, higher than the average value of the RCL and PCL, were determined as priorities. The LFM is shown in Figure 1. The LFM is a method of visually deriving priorities using a coordinate plane by marking the RCL on the horizontal axis and the average difference between the RCL and PCL on the vertical axis [12]; the median value on the horizontal axis is the average RCL, and the median value on the vertical axis is the average difference between the RCL and PCL. Generally, the first quadrant is the high-discrepancy, high-importance (HH) quadrant, where the difference between the 2 levels and demand for development is higher than the average (Figure 1). The third quadrant is the low-discrepancy, low-importance (LL) quadrant, where the difference between the 2 levels and demand for development is lower than the average; hence, items in the LL quadrant are not considered development priorities. Although the LFM makes it easy to prioritize items in the first quadrant, there is a need to ascertain where the next-priority quadrant is. Moreover, it may be difficult to determine priorities even within the same quadrant [9].

Finally, both Borich priority and the LFM were combined to determine the highest priority. The LFM is a simple quadrant plot, but when used with the Borich formula, it has the advantage of comprehensively considering the current status and future importance of training needs and the discrepancy between the 2. Specifically, if 10 needs fall into the HH quadrant in the LFM, we checked whether those 10 needs are also ranked as the top 10 priorities by the Borich formula. If so, those needs were considered to have the highest priority.

### Recruitment and Informed Consent

A recruitment notice introducing the study, including the research purpose, ethics protocol, and survey URL, was posted on a social networking site frequently visited by nursing students. Students who wished to participate completed a web-based survey in Google Forms. The first question required participants to confirm their informed consent to take part in the study. The form advised participants that they could withdraw at any time and that their data would be used only for security maintenance and research purposes. No personal information, such as names and email addresses, was collected to ensure participants’ anonymity.

### Ethics Approval

This study was approved by the Institutional Review Board of Wonkwang University (approval WKIRB-202011-SB-078).

## Results

### Participant Characteristics

A total of 222 nursing students participated in the survey, among whom women were the majority (n=201, 90.5% vs men, n=21, 9.5%), with an average age of 23.3 (SD 3.33) years. Most participants (n=95, 42.8%) were third-year students. Regarding the contents of VR programs experienced in real life, games were the most common (42/308, 13.6%), followed by movies or dramas (39/308, 12.7%). Unlike experience in real life, only 27 (N=222, 12.2%) students experienced VR in university subjects, with nursing-related subjects being the most frequent (20/44, 45.5%; Table 1).

**Table 1:** General characteristics of participants (n=222).

Characteristic		Value
**Sex (n=222), n (%)**		
	Male	21 (9.5)
	Female	201 (90.5)
Age (years), mean (SD)		23.3 (3.33)
**Year of university (n=222), n (%)**		
	Second year	36 (16.2)
	Third year	95 (42.8)
	Fourth year	91 (41)
**University location (n=222), n (%)**		
	Seoul capitol	50 (22.5)
	Metropolitan city	60 (27)
	Province	112 (50.5)
Nursing practice credits, mean (SD)		3.65 (1.5)
**VR^a^ experience contents (n=308, multiple responses), n (%)**		
	Game	42 (13.6)
	Movie or drama	39 (12.7)
	Education	34 (11)
	Travel or sightseeing	34 (11)
	Shopping	31 (10.1)
	Sports	17 (5.5)
	Concert	10 (3.2)
	Others	1 (0.3)
**VR experience during university curriculum (n=222), n (%)**		
	Yes	27 (12.2)
	No	195 (87.8)
**VR experience subjects at university (n=44, multiple responses), n (%)**		
	Nursing	20 (45.5)
	Engineering	8 (18.2)
	Media, architecture, or costume	5 (11.4)
	Art or music	5 (11.4)
	Pure science	3 (6.8)
	History	3 (6.8)
	English	0 (0)
	Others	0 (0)

^a^VR: virtual reality.

### Perception of VR

The overall perception of the VR program (calculated as the mean score for the 10 survey items) was 3.57 (SD 0.57), which was above average. Regarding the questionnaire items, the subjects showed the most positive response to the items indicating that the core nursing VR program would enhance clinical performance (mean 3.90, SD 0.98) and the quality of CNS (mean 3.90, SD 0.95). In contrast, the items “How much do you think you know about the concept of VR?”(mean 3.22, SD 0.90) and “Do you think VR products have become popular?” (mean 3.22, SD 0.92) had the lowest score but moderate-to-high levels of awareness (Table 2).

**Table 2:** Perception of virtual reality (VR) programs (N=222).

No	Items	Score, mean (SD)
1	Do you think that VR products have become popular?	3.22 (0.92)
2	How much do you think you know about the concept of VR?	3.22 (0.90)
3	Do you think that the nursing department needs to introduce VR programs?	3.60 (0.90)
4	Do you think that it is necessary to develop a VR program for teaching CNS^a^?	3.75 (0.90)
5	Do you think that the CNS VR program will enhance clinical performance such as imparting technical skills?	3.90 (0.98)
6	Do you think that the CNS VR program will help improve critical thinking?	3.40 (1.06)
7	Do you think that the CNS VR program will help improve communication?	3.35 (1.06)
8	Do you think that the CNS VR program will help in decision-making?	3.58 (1.09)
9	Do you think that the CNS VR program will enhance the quality of CNS lectures?	3.90 (0.95)
10	Do you think that the CNS VR program can replace face-to-face CNS lecture?	3.25 (1.21)
	Total	3.57 (0.57)

^a^CNS: core nursing skills.

### Priority of Demand for the Development of VR Programs for CNS: Results from the Borich Priority Formula

To find out the needs of nursing students for the development of the CNS VR program, the 2-tailed *t* test and Borich priority were calculated. The differences between the average PCL and RCL were considered statistically significant in the *t* test for all 20 CNS. As a result of calculating Borich priority, enema showed the highest demand for the development of a VR program (3.29), followed by administration of intramuscular (IM) injections (3.25) and tube feeding (2.91). In contrast, intravenous (IV) infusion (1.11), administration of oral medication (1.30), and administration of subcutaneous (SQ) injection (1.48) were considered the CNS with the lowest demand for the development of a VR program (Table 3).

**Table 3:** Results of paired 2-tailed t test and the Borich needs assessment model for the demand of the development of virtual reality (VR) programs (N=222).

No	Core nursing skills	PCL^a^, mean (SD)	RCL^b^, mean (SD)	Mean difference, mean (SD)	*t* test (*df*=221)	*P* value	Borich	
							Needs	Rank
1	Vital sign	3.69 (1.08)	4.19 (0.97)	0.50 (1.42)	5.20	<.001	2.08	12
2	Administration of oral medication	3.64 (0.93)	3.97 (1.12)	0.33 (1.29)	3.79	<.001	1.30	19
3	Administration of IM^c^ injection	3.73 (1.18)	4.45 (0.79)	0.73 (1.25)	8.69	<.001	3.25	2
4	Administration of SQ^d^ injection	3.99 (0.97)	4.33 (0.98)	0.34 (1.31)	3.89	<.001	1.48	18
5	Administration of ID^e^ injection	3.82 (1.09)	4.42 (0.87)	0.61 (1.27)	7.15	<.001	2.69	5
6	IV^f^ infusion	4.00 (1.08)	4.27 (1.19)	0.26 (1.51)	2.58	.011	1.11	20
7	Blood transfusion	3.90 (10.6)	4.42 (0.84)	0.53 (1.15)	6.84	<.001	2.33	9
8	Tube feeding	3.73 (1.09)	4.39 (0.95)	0.66 (1.25)	7.91	<.001	2.91	3
9	Urinary catheterization	3.93 (0.97)	4.35 (0.93)	0.42 (1.11)	5.63	<.001	1.82	16
10	Foley catheterization	3.93 (1.08)	4.34 (1.06)	0.41 (1.32)	4.62	<.001	1.78	17
11	Enema	3.65 (1.05)	4.40 (0.88)	0.75 (1.10)	10.16	<.001	3.29	1
12	Preoperative care	3.68 (1.03)	4.19 (0.92)	0.51 (1.20)	6.38	<.001	2.15	11
13	Postoperative care	3.77 (1.07)	4.37 (0.86)	0.60 (1.16)	7.67	<.001	2.62	6
14	Admission care	3.50 (1.03)	3.97 (1.21)	0.47 (1.40)	5.04	<.001	1.88	14
15	Gowning and gloving technique	3.72 (1.01)	4.18 (1.01)	0.46 (1.25)	5.46	<.001	1.92	13
16	Pulse oximeter and EKG^g^ monitoring	3.68 (1.06)	4.24 (0.98)	0.56 (1.27)	6.60	<.001	2.39	7
17	Supplying oxygen via nasal cannula	3.73 (1.06)	4.36 (0.93)	0.63 (1.16)	8.10	<.001	2.75	4
18	Endotracheal suction	3.80 (1.11)	4.34 (1.05)	0.54 (1.28)	6.30	<.001	2.34	8
19	Tracheostomy tube care	3.86 (1.04)	4.29 (1.06)	0.43 (1.23)	5.25	<.001	1.85	15
20	CPR^h^ and using defibrillator	3.96 (1.10)	4.45 (0.80)	0.50 (1.17)	6.30	<.001	2.21	10

^a^PCL: performance competence level.

^b^RCL: required competence level.

^c^IM: Intramuscular.

^d^SQ: subcutaneous.

^e^ID: intradermal.

^f^IV: Intravenous.

^g^EKG: electrocardiogram.

^h^CPR: cardiopulmonary resuscitation.

### Top-Priority Categories for the Demand for Developing VR Programs for CNS: Results From Combining Borich Priority and the LFM

**Figure 2:**
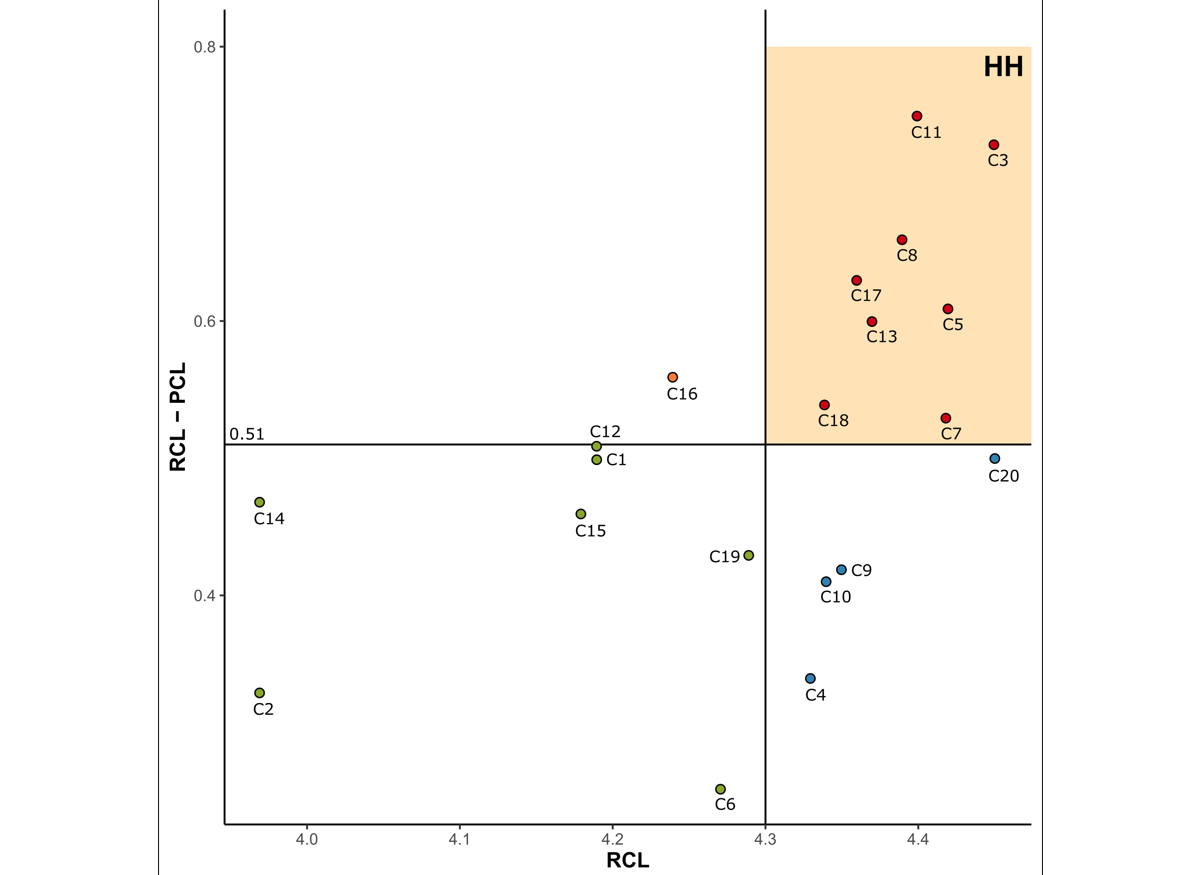
Visualization of priority of the development needs of a virtual reality program using the Locus for Focus Model. C: core nursing skill; HH: high-discrepancy, high-importance; PCL: present competency level; RCL: required competency level.

In the analysis of the priority for the development of a VR program using the LFM, the average RCL for the development of a VR program for CNS was 4.30, and the average level of the difference between the RCL and PCL was 0.51. As a result of dividing the coordinate planes using these averages as an axis, 8 items such as IM injection, ID injection, blood transfusion, tube feeding, enema, postoperative care, supplying oxygen via nasal cannula, and endotracheal suction were included in the HH quadrant (Figure 2).

Finally, both Borich priority and the LFM were combined to determine the highest priority. In other words, any number of the 8 items of the first quadrant (HH) using the LFM and the 8 items of the Borich ranking priority could be selected. As a result, 7 items were included in the top priority for development, including IM injection, ID injection, tube feeding, enema, postoperative care, supplying oxygen via nasal cannula, and endotracheal suction (Table 4). All but 1 of the 7 items (supplying oxygen via nasal cannula) were found to correspond to a moderate or higher level of difficulty set by KABONE, and 2 of them (ID injection and endotracheal suction) were found to correspond to a high level of difficulty.

**Table 4:** Top-priority developmental needs for core nursing skills according to Borich needs assessment model and the Locus for Focus Model (LFM).

No	Core nursing skill	Borich rank	LFM quadrant	Top priority
1	Vital sign	12	LL^a^	
2	Administration of oral medication	19	LL	
3	Administration of IM^b^ injection	2	HH^c^	✓^d^
4	Administration of SQ^e^ injection	18	LH^f^	
5	Administration of ID^g^ injection	5	HH	✓
6	IV^h^ infusion	20	LL	
7	Blood transfusion	9	HH	
8	Tube feeding	3	HH	✓
9	Urinary catheterization	16	LH	
10	Foley catheterization	17	LH	
11	Enema	1	HH	✓
12	Preoperative care	11	LL	
13	Postoperative care	6	HH	✓
14	Admission care	14	LL	
15	Gowning and gloving technique	13	LL	
16	Pulse oximeter and EKG^i^ monitoring	7	HL^j^	
17	Supplying oxygen via nasal cannula	4	HH	✓
18	Endotracheal suction	8	HH	✓
19	Tracheostomy tube care	15	LL	
20	CPR^k^ and using defibrillator	10	LH	

^a^LL: low discrepancy, low importance.

^b^IM: intramuscular.

^c^HH: high discrepancy, high importance.

^d^✓: indicates the highest priority for developing into virtual reality programs.

^e^SQ: subcutaneous.

^f^LH: low discrepancy, high importance.

^g^ID: intradermal.

^h^IV: intravenous.

^i^EKG: electrocardiogram.

^j^HL: high discrepancy, low importance.

^k^CPR: cardiopulmonary resuscitation.

## Discussion

### Principal Findings

The purpose of this study was to use the Borich priority formula and LFM to understand which CNS most require the development of VR programs to meet the needs of nursing students.

The study participants comprised 222 students in their second year or higher, who had taken courses on fundamental nursing or CNS and had experienced VR programs. Although 72.8% of the participants experienced VR programs in their daily lives, only 12.2% used them in the university curriculum. The participants showed the lowest score (mean 3.22, SD 0.90) for the questionnaire item regarding the concept of VR. This appeared to be consistent with the previous research results, which indicated that only 61.6% of students who had heard of VR or augmented reality (AR) before (99.7%) said they could accurately distinguish between VR and AR [13]. Nevertheless, the participants responded that VR programs for CNS would help enhance clinical performance (mean 3.90, SD 0.98), as well as the quality of CNS education (mean 3.90, SD 0.95), showing positive expectations for practical training using VR programs in the future. This was consistent with the results of previous related studies, which stated that VR- and AR-applied education would enhance learning and clinical performance [13]. In addition, the use of VR programs not only improves students’ understanding and proficiency in CNS but also allows them to experience, in advance, how to respond to various patient reactions when directly performing CNS in real nursing situations [14]. Training using VR programs that provided repetitive training under the supervision of the educator increased the skill-retaining period compared with traditional one-time face-to-face training or self-practice in an open laboratory without appropriate feedback from the educator [15,16]. These prior research results are consistent with the results of this study, which indicate that education using VR programs will help improve clinical performance in hospitals in the future.

Based on the results of this study, the participants identified the differences between the PCL and RCL in developing VR programs for CNS. In other words, the PCL was statistically significantly lower than the RCL for all the items. This result means participants showed awareness of the need to develop VR programs when there is a difference between the PCL and RCL [17]. The top development priority items according to Borich needs formula included enema (3.29), administration of IM injection (3.25), and tube feeding (2.91). On the other hand, the lowest rankings were IV infusion (1.11), administration of oral medication [1.30], and administration of SQ injection [18]. In the coordinate plane using the LFM, the priority items for development (HH quadrant) totaled 8 items, including IM injection, ID injection, blood transfusion, tube feeding, etc. The KABONE classifies 20 CNS into higher (6 skills), middle (9 skills), and lower (5 skills) levels of difficulty. The LFM analysis revealed that 3 of 8 items in the HH quadrant (ID injection, blood transfusion, and endotracheal suction) corresponded to higher-difficulty CNS according to the KABONE. Additionally, 7 items in the LL quadrant (vital sign, administration of oral medication, IV infusion, preoperative care, admission care, gowning and gloving technique, and tracheostomy tube care) were not considered priority items for the development of a VR program, whereas 3 items (vital sign, oral medication, and admission care) in that quadrant were classified as lower difficulty according to the KABONE. In other words, the difficulty of CNS set by the KABONE and priority in the development of a VR program by the LFM according to the results of this study were consistent.

In the last step, the items and number of items in the HH quadrant of the LFM, as well as the same number of priority items according to the Borich needs assessment, were identified to determine the overlapping items in the 2 methods. As a result of the analysis, a total of 7 items, including IM injection, ID injection, tube feeding, enema, postoperative care, supplying oxygen via nasal cannula, and endotracheal suction, were found to be the top-priority items for the development of a VR program.

This result was similar with the items identified in previous studies, indicating that new graduate nurses and nursing students had the lowest confidence in the necessary skills for enemas, tracheostomy tube care, tube feeding, postoperative care, etc [18-23]. Additionally, nursing students chose tracheostomy tube care and enema as the CNS that they did not have the opportunity to practice in person or observe. This finding means that most of the clinical practice of nursing students was focused on noninvasive, safe, and simple skills that do not invade patient’s privacy, involving low exposure of the patient’s body. In other words, the participants were found to have selected invasive items with few opportunities for direct execution and observation during practical apprenticeship training, despite their close association with patient safety and importance, as CNS of high priority for the development of a VR program. This finding can also be confirmed in that most of the skills belonging to LL quadrant in the LFM, an area that is not considered for development priorities, are skills that can be delivered verbally rather than through direct contact with patients.

Previous related studies indicated that students who undergo CNS training through a VR program were able to minimize possible harm to patients when exposed to the actual clinical environment [24,25]. VR programs are designed to prevent the student from proceeding to the next step if they make mistakes related to patient safety or choose the wrong method; this may have contributed to the students improving their skills. Therefore, the development of VR programs for invasive CNS should be prioritized to reduce the possibility of the occurrence of errors during clinical practice and the fear of students to learn skills that can harm patients through repetitive practice of the nursing skills in a safe and realistic environment. CNS are mostly taught in a preparatory course before clinical practice. Nursing students, therefore, want a program that allows for effective practice of CNS as a preparatory course in school [26]. Moreover, most of the items that necessitate the development of VR programs show a low frequency of indirect experience in practical apprenticeship training and in-school simulation practice [22]. Accordingly, the reinforcement of skill learning through VR programs for these items appears to be necessary.

Education comprises 3 elements: the educator, student, and educational content. The “student” variable is considered the most important factor in determining the quality of educational performance. Students are the end consumers of all the content provided in the curriculum and are substantially influenced in terms of their career paths through the enhancement of their abilities and competencies. Therefore, it is essential to first analyze the needs of learners and configure the curriculum accordingly in the new, normal post–COVID-19 era. Particularly, in the field of nursing, where practical education occupies a large part of the curriculum, it is necessary to identify the needs of students for practical education and develop a curriculum that reflects them [26]. Post–COVID-19 education requires a paradigm shift. In other words, it is necessary to establish an on-demand form of education that reflects the diverse competencies and needs of students [1]. In this respect, this study’s analysis of the demands for the development of VR programs for teaching CNS is important. In other words, it will be necessary to consider various approaches to teach the 20 CNS to further provide learner-centered personalized education, hence meeting the needs of students by applying traditional learning methods to the items that generate low-development demands and developing VR programs for items that generate high-development demands.

### Limitations

This study has the following limitations. First, in terms of study participants and sampling, the survey was conducted on a website visited by many nursing students. Therefore, it was not possible to perform more precise sampling by region and year of university. This limits generalization in the interpretation and application of the research results. Therefore, there is need for future research to increase the possibility of generalization through sampling by region and year of university. Second, there is a need for further research according to demographic characteristics, such as how participants take relevant classes (face to face or remotely) or their exposure to clinical or VR training. Previous studies on confidence in CNS showed differences in difficulty and confidence depending on the general characteristics of the subjects. In other words, conducting research by considering the variables more comprehensively according to the demographic characteristics of participants, such as how they take CNS-related courses, will enable the development of more practical and systematic VR programs for teaching CNS, providing appropriate content for students in each year of university. Third, this study was conducted among nursing students. Therefore, there is a need to conduct studies with participants with various experiences, such as new nurses or preceptors. In other words, it will be possible to establish a continuous and virtuous cycle of learning that connects schools to hospitals and back to schools by examining the training methods and confidence in CNS for new nurses and reflecting these results back into the curriculum of the university.

### Comparison With Prior Work

There are many studies on CNS, but there are no studies examining the needs of students following the development of CNS VR programs. As there are no studies similar to this study, it is difficult to compare its results with those of previous studies.

### Conclusions

Nursing education is aimed at acquiring and integrating knowledge, attitudes, and skills to solve health problems through lectures and practice. CNS are practical skills that each individual nursing student must acquire through repeated practice before clinical practice, requiring self-directed learning capabilities. The analysis of the development demands and priorities for developing VR programs for CNS showed that nursing students generally asked for the development of VR programs for nursing skills that involved invasive procedures. The results of this study are intended to help various aspects of in-school practical education using VR programs in nursing departments, which are currently facing difficulties in teaching CNS on the web owing to COVID-19.

## Abbreviations

AR: augmented reality
CNS: core nursing skills
CVI: content validity index
HH: high discrepancy, high importance
I-CVI: Item–content validity index
ID: intradermal
IM: intramuscular
IV: intravenous
KABONE: Korean Accreditation Board of Nursing Education
LFM: Locus for Focus Model
LL: low discrepancy, low importance
LNA: learning needs analysis
PCL: present competency level
RCL: required competency level
SQ: subcutaneous
VR: virtual reality
